# Supplementary material for: Elevated Serum Gastrin Is Associated with Melanoma Progression: Putative Role in Increased Migration and Invasion of Melanoma Cells
Source: Int J Mol Sci. 2023 Nov 28;24(23):16851. doi: 10.3390/ijms242316851 (PMC10706711; doi:10.3390/ijms242316851)
Supplement: Supplementary file 1 [file ijms-24-16851-s001.zip › Figure S1.pdf]

A

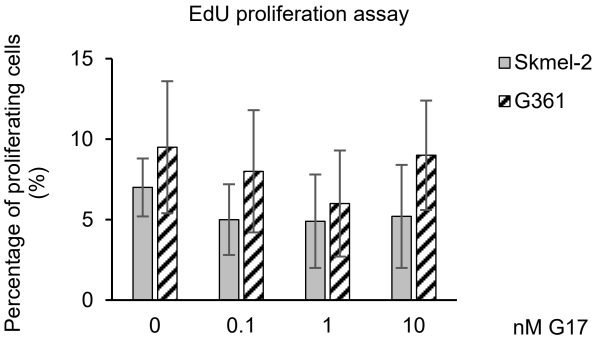

B

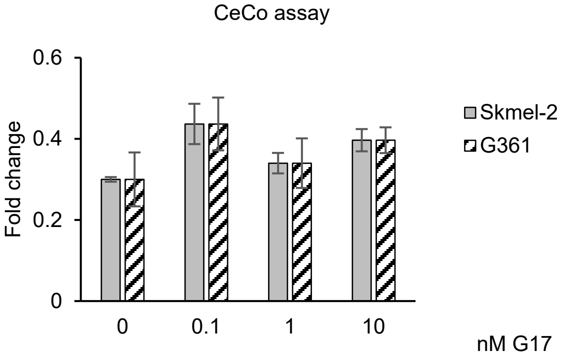

C

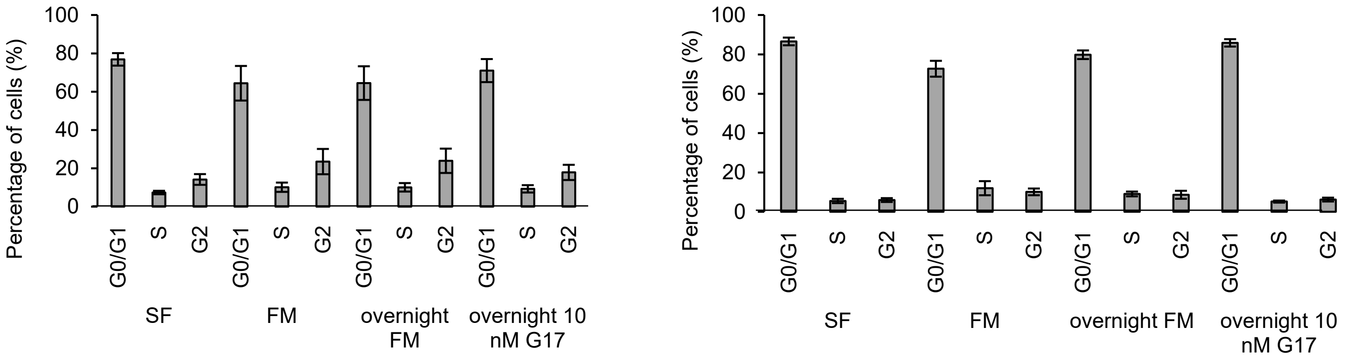

Figure S1. Gastrin has no effect on melanoma cell proliferation. Gastrin treatment covering a 100-fold range had no significant effect on the percentage of EdU labelled cells (A) or cellular biomass (B). Flow cytometry revealed no changes in the proportion of cells in different stages of the cell cycle when treated with gastrin. Cancer cells in the control group were kept in serum free (SF) medium; in the positive control group cells were incubated in full medium (FM); in the overnight-FM group cells were treated with supplemented medium for 8 hours; while cell in overnight-G17 group were stimulated with 10 nM gastrin for 8 hours prior to cell cycle analysis (C, left, SK-MEL-2; right, G-361).
