# Supplementary material for: Elevated Serum Gastrin Is Associated with Melanoma Progression: Putative Role in Increased Migration and Invasion of Melanoma Cells
Source: Int J Mol Sci. 2023 Nov 28;24(23):16851. doi: 10.3390/ijms242316851 (PMC10706711; doi:10.3390/ijms242316851)
Supplement: Supplementary file 1 [file ijms-24-16851-s001.zip › Figure S2.pdf]

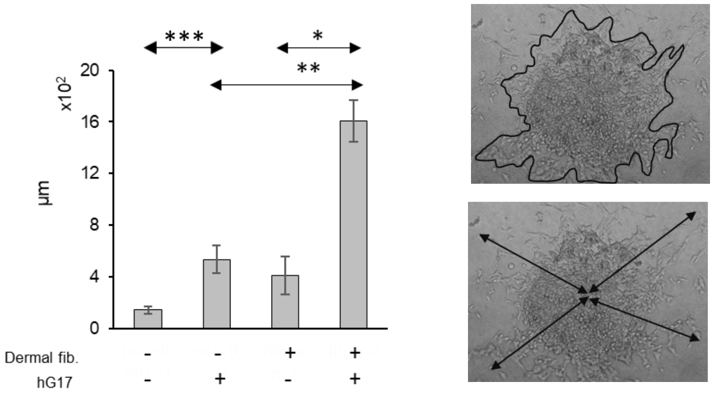


**Figure S2.** Gastrin stimulates melanoma invasion in the presence of fibroblasts. Gastrin markedly increased the distance of individual melanoma cells from spheroid centre if surrounded by stromal cells. Y-axis indicates distance of invaded tumour cells in μm from spheroid core.
