# Supplementary material for: Elevated Serum Gastrin Is Associated with Melanoma Progression: Putative Role in Increased Migration and Invasion of Melanoma Cells
Source: Int J Mol Sci. 2023 Nov 28;24(23):16851. doi: 10.3390/ijms242316851 (PMC10706711; doi:10.3390/ijms242316851)
Supplement: Supplementary file 1 [file ijms-24-16851-s001.zip › Table S1.pdf]

Table S1. Demographics of melanoma and basal cell cancer patient cohorts

	**Melanoma**	**Basal cell cancer**
Total	89	26
Male/Female	39/50	16/10
Mean age [year]	60.6±1.6	76.0±2.2
Min/Max. age [year]	25/89	54/93
Acid inhibitor usage [year]	3.9±0.9	4.22±0.45
PPI/H2RA[1]	16/5	13/2
Hp +ve/-ve[2]	37/51	16/10

[1]proton pump inhibitor, histamine type 2 receptor antagonists

[2]Helicobacter pylori
