# Supplementary material for: Elevated Serum Gastrin Is Associated with Melanoma Progression: Putative Role in Increased Migration and Invasion of Melanoma Cells
Source: Int J Mol Sci. 2023 Nov 28;24(23):16851. doi: 10.3390/ijms242316851 (PMC10706711; doi:10.3390/ijms242316851)
Supplement: Supplementary file 1 [file ijms-24-16851-s001.zip › Table S2.pdf]

A

No.:	Description	Accession	Prot	Unique Pep.	Pep.	Heavy/ Light	AAs	MW [kDa]
**1**	*CD166 antigen (CD166)*	Q13740	1	1	1	2.193	583	65.1
**2**	*Prosaposin (SAP)*	P07602	1	4	4	1.907	524	58.1
**3**	*72 kDa type IV collagenase (MMP-2)*	P08253	1	7	7	1.495	660	73.8
**4**	*Glia-derived nexin (GDN)*	P07093	1	10	11	0.941	398	44
**5**	*Sushi repeat-containing protein SRPX (SRPX)*	P78539	1	8	9	0.939	464	51.5
**6**	*Calsyntenin-1 (CSTN1)*	O94985	2	15	16	0.939	981	109.7
**7**	*Dystroglycan (DAG1)*	Q14118	1	9	9	0.863	895	97.4
**8**	*Fibronectin (FINC)*	P02751	1	59	63	0.863	2386	262.5
**9**	*Neurosecretory protein VGF (VGF)*	O15240	1	14	15	0.795	615	67.2
**10**	*Cystatin-C (CYTC)*	P01034	2	4	5	0.782	146	15.8
**11**	*Protein NOV homolog (NOV)*	P48745	1	10	13	0.777	357	39.1
**12**	*Metalloproteinase inhibitor 2 (TIMP2)*	P16035	1	10	12	0.705	220	24.4
**13**	*Insulin-like growth factor-binding protein 7 (IBP7)*	Q16270	2	14	15	0.676	282	29.1
**14**	*Insulin-like growth factor-binding protein 2 (IBP2)*	P18065	1	9	10	0.674	325	34.8
**15**	*Cadherin-2 (CADH2)*	P19022	1	2	2	0.655	906	99.7
**16**	*SPARC (SPRC)*	P09486	1	3	4	0.648	303	34.6
**17**	*Tumor necrosis factor-inducible gene 6 protein (TSG6)*	P98066	1	2	2	0.628	277	31.2
**18**	*Sulfhydryl oxidase 1 (QSOX1)*	O00391	1	7	7	0.626	747	82.5
**19**	*Thrombospondin-1 (TSP1)*	P07996	1	22	23	0.613	1170	129.3
**20**	*Tissue-type plasminogen activator (TPA)*	P00750	1	7	8	0.612	562	62.9
**21**	*Melanoma-derived growth regulatory protein (MIA)*	Q16674	1	3	3	0.594	131	14.5
**22**	*Nidogen-1 (NID1)*	P14543	2	13	14	0.559	1247	136.3
**23**	*Complement factor I (CFAI)*	P05156	1	1	1	0.555	583	65.7
**24**	*Lactadherin (MFGM)*	Q08431	1	1	1	0.549	387	43.1
**25**	*Neuroblastoma suppressor of tumorigenicity 1 (NBL1)*	P41271	1	1	1	0.527	181	19.4
**26**	*Insulin-like growth factor-binding protein 3 (IBP3)*	P17936	1	4	4	0.501	291	31.7
**27**	*Follistatin-related protein 1 (FSTL1)*	Q12841	1	4	6	0.496	308	35
**28**	*Tenascin (TENA)*	P24821	2	51	57	0.462	2201	240.7
**29**	*Lysyl oxidase homolog 2 (LOXL2)*	Q9Y4K0	1	7	7	0.461	774	86.7
**30**	*Granulocyte-macrophage colony-stimulating factor receptor subunit alpha (CSF2R)*	P15509	1	2	2	0.454	400	46.2
**31**	*Disintegrin and metalloproteinase domain-containing protein 9 (ADAM9)*	Q13443	1	3	3	0.452	819	90.5
**32**	*Tumor necrosis factor receptor superfamily member 12A (TNR12)*	Q9NP84	1	1	1	0.381	129	13.9
**33**	*Peptidyl-glycine alpha-amidating monooxygenase (AMD)*	P19021	1	3	3	0.376	973	108.3
**34**	*Galectin-3-binding protein (LG3BP)*	Q08380	1	9	10	0.366	585	65.3
**35**	*Laminin subunit alpha-4 (LAMA4)*	Q16363	1	21	25	0.361	1823	202.4
**36**	*Protein disulfide-isomerase A4 (PDIA4)*	P13667	5	14	16	0.337	645	72.9
**37**	*Collagen alpha-1(XII) chain (COCA1)*	Q99715	1	34	34	0.324	3063	332.9
**38**	*Matrilin-2 (MATN2)*	O00339	1	10	14	0.318	956	106.8
**39**	*Extracellular matrix protein 1 (ECM1)*	Q16610	1	7	7	0.273	540	60.6
**40**	*Cathepsin L1 (CATL1)*	P07711	1	3	3	0.271	333	37.5
**41**	*Apolipoprotein D (APOD)*	P05090	1	3	3	0.245	189	21.3
**42**	*Serine protease 23 (PRS23)*	O95084	1	2	3	0.221	383	43
**43**	*Beta-2-microglobulin (B2MG)*	P61769	1	2	3	0.218	119	13.7
**44**	*Laminin subunit gamma-1 (LAMC1)*	P11047	1	30	34	0.198	1609	177.5
**45**	*Matrix metalloproteinase-14 (MMP14)*	P50281	1	1	1	0.191	582	65.9
**46**	*Protein disulfide-isomerase A3 (PDIA3)*	P30101	1	20	20	0.19	505	56.7
**47**	*Protein disulfide-isomerase (PDIA1)*	P07237	1	6	7	0.175	508	57.1
**48**	*Disintegrin and metalloproteinase domain-containing protein 10 (ADA10)*	O14672	1	4	4	0.173	748	84.1

**49**	*Laminin subunit beta-1 (LAMB1)*	P07942	1	19	19	0.15	1786	197.9
**50**	*Cathepsin D (CATD)*	P07339	1	2	2	0.15	412	44.5
**51**	*Vascular endothelial growth factor receptor 1 (VGFR1)*	P17948	1	5	5	0.142	1338	150.7
**52**	*Trans-Golgi network integral membrane protein 2 (TGON2)*	O43493	1	5	5	0.115	480	51.1
**53**	*LDLR chaperone MESD (MESD)*	Q14696	1	4	5	0.112	234	26.1
**54**	*Connective tissue growth factor (CTGF)*	P29279	1	26	26	0.112	349	38.1
**55**	*Multiple inositol polyphosphate phosphatase 1 (MINP1)*	Q9UNW1	1	1	1	0.084	487	55
**56**	*Collagen triple helix repeat-containing protein 1 (CTHR1)*	Q96CG8	1	5	6	0.079	243	26.2
**57**	*Nucleobindin-1 (NUCB1)*	Q02818	2	12	15	0.054	461	53.8
**58**	*Granulins (GRN)*	P28799	1	5	8	0.054	593	63.5
**59**	*Calumenin (CALU)*	O43852	1	4	4	0.053	315	37.1
**60**	*Growth-regulated alpha protein (GROA)*	P09341	17	3	6	0.044	107	11.3
**61**	*45 kDa calcium-binding protein (CAB45)*	Q9BRK5	1	10	10	0.037	362	41.8
**62**	*Calreticulin (CALR)*	P27797	1	1	1	0.029	417	48.1
**63**	*Cell surface glycoprotein MUC18 (MUC18)*	P43121	5	12	13	0.029	646	71.6
**64**	*Glucosidase 2 subunit beta (GLU2B)*	P14314	1	12	13	0.014	528	59.4
**65**	*Interleukin-8 (IL8)*	P10145	1	4	5	0.012	99	11.1

B

No.:	Description	Accession	Prot.	Unique Pep.	Pep.	Heavy/ Light	AAs	MW [kDa]
**1**	*Prosaposin (SAP)*	P07602	1	7	8	1.785	524	58.1
**2**	*Neurosecretory protein VGF (VGF)*	O15240	1	34	34	0.933	615	67.2
**3**	*Glia-derived nexin (GDN)*	P07093	1	4	4	0.917	398	44
**4**	*72 kDa type IV collagenase (MMP-2)*	P08253	1	17	17	0.9	660	73.8
**5**	*Metalloproteinase inhibitor 1 (TIMP1)*	P01033	1	5	5	0.87	207	23.2
**6**	*Granulins (GRN)*	P28799	1	15	16	0.839	593	63.5
**7**	*Dystroglycan (DAG1)*	Q14118	1	12	12	0.811	895	97.4
**8**	*Sushi repeat-containing protein SRPX (SRPX)*	P78539	1	9	9	0.791	464	51.5
**9**	*Calsyntenin-1 (CSTN1)*	O94985	2	17	18	0.771	981	109.7
**10**	*Insulin-like growth factor-binding protein 2 (IBP2)*	P18065	1	15	16	0.761	325	34.8
**11**	*Stanniocalcin-1 (STC1)*	P52823	1	2	2	0.734	247	27.6
**12**	*Metalloproteinase inhibitor 2 (TIMP2)*	P16035	1	14	16	0.7	220	24.4
**13**	*Insulin-like growth factor-binding protein 3 (IBP3)*	P17936	20	13	13	0.695	291	31.7
**14**	*Fibronectin (FINC)*	P02751	3	78	78	0.683	2386	262.5
**15**	*Cadherin-2 (CADH2)*	P19022	1	1	1	0.675	906	99.7
**16**	*Sulfhydryl oxidase 1 (QSOX1)*	O00391	1	5	5	0.661	747	82.5
**17**	*ADM (ADML)*	P35318	1	5	6	0.647	185	20.4
**18**	*Cathepsin L2 (CATL2)*	O60911	2	1	3	0.638	334	37.3
**19**	*Cadherin-1 (CADH1)*	P12830	1	1	1	0.628	882	97.4
**20**	*Protein NOV homolog (NOV)*	P48745	1	12	13	0.619	357	39.1
**21**	*Cystatin-C (CYTC)*	P01034	2	6	6	0.608	146	15.8
**22**	*Melanoma-derived growth regulatory protein (MIA)*	Q16674	1	5	5	0.606	131	14.5
**23**	*Testican-1 (TICN1)*	Q08629	2	8	9	0.599	439	49.1
**24**	*Follistatin-related protein 5 (FSTL5)*	Q8N475	2	3	3	0.585	847	95.7
**25**	*SPARC (SPRC)*	P09486	1	5	6	0.572	303	34.6
**26**	*Cell migration-inducing and hyaluronan-binding protein (CEMIP)*	Q8WUJ3	1	7	9	0.553	1361	152.9
**27**	*Tenascin (TENA)*	P24821	2	24	27	0.544	2201	240.7
**28**	*Amyloid beta A4 protein (A4)*	P05067	2	11	12	0.534	770	86.9
**29**	*Insulin-like growth factor-binding protein 7 (IBP7)*	Q16270	1	12	12	0.532	282	29.1
**30**	*Peptidyl-glycine alpha-amidating monooxygenase (AMD)*	P19021	1	10	11	0.528	973	108.3
**31**	*Tissue-type plasminogen activator (TPA)*	P00750	1	10	11	0.514	562	62.9
**32**	*Inter-alpha-trypsin inhibitor heavy chain H5 (ITIH5)*	Q86UX2	1	9	10	0.502	942	104.5
**33**	*Neuroblastoma suppressor of tumorigenicity 1 (NBL1)*	P41271	1	3	3	0.499	181	19.4
**34**	*Collagen alpha-3(V) chain (CO5A3)*	P25940	1	2	3	0.489	1745	172
**35**	*Laminin subunit alpha-1 (LAMA1)*	P25391	2	48	51	0.487	3075	336.9
**36**	*Amyloid-like protein 2 (APLP2)*	Q06481	1	2	3	0.477	763	86.9
**37**	*Follistatin-related protein 1 (FSTL1)*	Q12841	1	8	9	0.468	308	35
**38**	*Serine protease 23 (PRS23)*	O95084	1	3	4	0.467	383	43
**39**	*EMILIN-2 (EMIL2)*	Q9BXX0	1	6	8	0.445	1053	115.6
**40**	*Transmembrane glycoprotein NMB (GPNMB)*	Q14956	1	3	4	0.426	572	63.9
**41**	*Serine protease HTRA1 (HTRA1)*	Q92743	1	12	13	0.425	480	51.3
**42**	*Tartrate-resistant acid phosphatase type 5 (PPA5)*	P13686	1	2	2	0.391	325	36.6
**43**	*Apolipoprotein D (APOD)*	P05090	1	4	5	0.39	189	21.3
**44**	*Lactadherin (MFGM)*	Q08431	1	1	1	0.37	387	43.1
**45**	*Vitamin K-dependent protein S (PROS)*	P07225	1	5	5	0.353	676	75.1
**46**	*Extracellular matrix protein 1 (ECM1)*	Q16610	1	16	16	0.351	540	60.6
**47**	*Laminin subunit gamma-1 (LAMC1)*	P11047	1	43	46	0.343	1609	177.5

**48**	*Angiopoietin-related protein 2 (ANGL2)*	Q9UKU9	2	5	5	0.341	493	57.1
**49**	*Matrix metalloproteinase-14 (MMP14)*	P50281	1	6	6	0.341	582	65.9
**50**	*Cathepsin D (CATD)*	P07339	1	7	8	0.313	412	44.5
**51**	*Acid ceramidase (ASAH1)*	Q13510	1	4	4	0.309	395	44.6
**52**	*Galectin-3-binding protein (LG3BP)*	Q08380	1	7	8	0.307	585	65.3
**53**	*Collagen alpha-1(VI) chain (CO6A1)*	P12109	1	1	1	0.262	1028	108.5
**54**	*Thrombospondin-1 (TSP1)*	P07996	1	11	11	0.258	1170	129.3
**55**	*Cathepsin L1 (CATL1)*	P07711	2	9	11	0.228	333	37.5
**56**	*Laminin subunit alpha-4 (LAMA4)*	Q16363	1	6	9	0.221	1823	202.4
**57**	*Growth-regulated alpha protein (GROA)*	P09341	19	5	6	0.221	107	11.3
**58**	*Laminin subunit beta-1 (LAMB1)*	P07942	3	26	29	0.213	1786	197.9
**59**	*Immunoglobulin superfamily member 8 (IGSF8)*	Q969P0	1	8	8	0.198	613	65
**60**	*Beta-2-microglobulin (B2MG)*	P61769	1	2	2	0.197	119	13.7
**61**	*Protein jagged-1 (JAG1)*	P78504	1	3	3	0.193	1218	133.7
**62**	*Collagen alpha-2(IV) chain (CO4A2)*	P08572	1	13	13	0.173	1712	167.4
**63**	*Growth/differentiation factor 15 (GDF15)*	Q99988	1	13	14	0.166	308	34.1
**64**	*DnaJ homolog subfamily B member 11 (DJB11)*	Q9UBS4	1	2	2	0.156	358	40.5
**65**	*Nucleobindin-1 (NUCB1)*	Q02818	14	26	30	0.155	461	53.8
**66**	*Calnexin (CALX)*	P27824	1	3	4	0.153	592	67.5
**67**	*Alpha-2-macroglobulin (A2MG)*	P01023	1	2	4	0.152	1474	163.2
**68**	*Kunitz-type protease inhibitor 1 (SPIT1)*	O43278	1	1	1	0.15	529	58.4
**69**	*Receptor-type tyrosine-protein phosphatase S (PTPRS)*	Q13332	1	10	11	0.144	1948	216.9
**70**	*Protein disulfide-isomerase A4 (PDIA4)*	P13667	5	23	24	0.141	645	72.9
**71**	*Disintegrin and metalloproteinase domain-containing protein 10 (ADA10)*	O14672	1	9	9	0.134	748	84.1
**72**	*Metalloproteinase inhibitor 3 (TIMP-3)*	P35625	1	7	7	0.125	211	24.1
**73**	*Protein disulfide-isomerase A3 (PDIA3)*	P30101	1	21	22	0.11	505	56.7
**74**	*Melanocyte protein PMEL (PMEL)*	P40967	1	7	8	0.093	661	70.2
**75**	*Nucleobindin-2 (NUCB2)*	P80303	1	5	7	0.091	420	50.2
**76**	*Golgi apparatus protein 1 (GSLG1)*	Q92896	1	12	12	0.091	1179	134.5
**77**	*Multiple inositol polyphosphate phosphatase 1 (MINP1)*	Q9UNW1	1	1	1	0.089	487	55
**78**	*78 kDa glucose-regulated protein (GRP78)*	P11021	3	31	36	0.073	654	72.3
**79**	*Protein CYR61 (CYR61)*	O00622	1	9	11	0.071	381	42
**80**	*Trans-Golgi network integral membrane protein 2 (TGON2)*	O43493	1	6	7	0.069	480	51.1
**81**	*45 kDa calcium-binding protein (CAB45)*	Q9BRK5	1	9	11	0.067	362	41.8
**82**	*Cathepsin B (CATB)*	P07858	1	9	9	0.06	339	37.8
**83**	*Chondroitin sulfate proteoglycan 4 (CSPG4)*	Q6UVK1	1	21	23	0.055	2322	250.4
**84**	*Collagen triple helix repeat-containing protein 1 (CTHR1)*	Q96CG8	1	4	5	0.053	243	26.2
**85**	*Midkine (MK)*	P21741	1	3	4	0.052	143	15.6
**86**	*Procollagen-lysine,2-oxoglutarate 5-dioxygenase 3 (PLOD3)*	O60568	1	9	13	0.048	738	84.7
**87**	*Calreticulin (CALR)*	P27797	1	6	8	0.04	417	48.1
**88**	*Chitinase domain-containing protein 1 (CHID1)*	Q9BWS9	1	2	3	0.035	393	44.9
**89**	*Neural cell adhesion molecule L1 (L1CAM)*	P32004	2	8	8	0.031	1257	139.9
**90**	*Calumenin (CALU)*	O43852	1	10	10	0.024	315	37.1
**91**	*Polypeptide N-acetylgalactosaminyltransferase 2 (GALT2)*	Q10471	1	15	15	0.023	571	64.7
**92**	*Cell surface glycoprotein MUC18 (MUC18)*	P43121	2	16	16	0.02	646	71.6
**93**	*Glucosidase 2 subunit beta (GLU2B)*	P14314	1	11	11	0.011	528	59.4

Table S2. List of classically secreted isotope labelled proteins with signal peptide sequence showing alteration after gastrin treatment characterized from Skmel-2 (A) and G361 (B) secretome. Columns from left to right contain protein name, protein identification accession number based on the FASTA database, number of identified proteins in the protein group of a master protein (#prot), number of peptide sequences unique to the protein group (#unique

pep), number of distinct peptide sequences in the protein group (#pep), ratio of labelled to unlabelled proteins (#H/L), the sequence length of the protein (#AA) and the calculated molecular weight of the protein (#MW).
